# Phlegmasia Alba Dolens Complicating Rhabdomyolysis

**DOI:** 10.7759/cureus.14080

**Published:** 2021-03-24

**Authors:** Leonardo P Suciadi, Aloysius N Aristo

**Affiliations:** 1 Cardiology, Siloam Hospitals Kebon Jeruk/Siloam Heart Institute, Jakarta, IDN; 2 Cardiology, Mitra Keluarga Kalideres Hospital, Jakarta, IDN

**Keywords:** phlegmasia, phlegmasia alba dolens, massive dvt, rhabdomyolysis, proximal massive deep vein thrombosis, acute kidney injury, deep vein thrombosis, dvt

## Abstract

A 57-year-old woman with obesity, severe dyslipidemia and hypertension presented with acute onset of excruciating pain at left leg followed by entire leg swelling without a specific provocation. Physical examination revealed a significant left lower limb oedema along with pale skin, livedo reticularis, poikilothermia, and diminished distal artery pulsation. Urgent vascular Doppler sonography of left lower limb showed obstructive venous thrombus and non-compressible vessel at level of iliofemoral vein. Arterial systems were without any obliteration but with relatively reduced flow to distal part. Blood test resulted in significantly raised creatinine and creatine kinase (CK) level. Diagnosis of phlegmasia alba dolens with complication of rhabdomyolysis and acute kidney injury had been made initially. The patient was treated with heparin as well as rehydration using saline solution and bicarbonate. Eventually, she had clinical improvement during hospitalization and been discharged with resolution of creatinine and CK level.

## Introduction

Phlegmasia dolens is a rare disorder caused by massive deep venous thrombosis (DVT) at proximal limbs. It is clinically classified as phlegmasia cerulea dolens (PCD) and phlegmasia alba dolens (PAD) [1]. The differences between PAD and PCD are the amount of deoxyhemoglobin in the venous plexus of the skin and subcutaneous tissue and the degree of discomfort produced by the associated venous hypertension and elevated compartment pressures [2]. Phlegmasia alba dolens (white leg) is characterized by swollen, pale, and painful limb. This condition occurs when venous thrombosis progresses to massive occlusion of the major deep venous system of the leg (e.g., at iliofemoral vein) with sparing of the collateral veins to remove subdermal and dermal venous blood before cyanosis occurs [3].

Haimovici described PAD as a stage in the thrombophlebitic continuum prior to PCD [4]. PCD is characterized by limb swelling, significant cyanosis, and severe pain [3]. In PCD, the thrombosis also extends to collateral veins resulting in tremendous venous congestion accompanied by massive fluid sequestration and more significant oedema [5]. This condition leads to systemic hypovolemia in rapid progression if not treated well. PCD may progress to irreversible venous gangrene in 40-60% cases unless it is treated in an early phase [6]. PCD have mortality rates of 25% to 40% and amputation rates of 20% to 50%. As of now, there are still a few publications about phlegmasia alba dolens. Specific therapeutic algorithms and guidelines are still lacking. Physicians might still have limited knowledge and experience of this life-threatening condition [6].

## Case presentation

**Figure 1:**
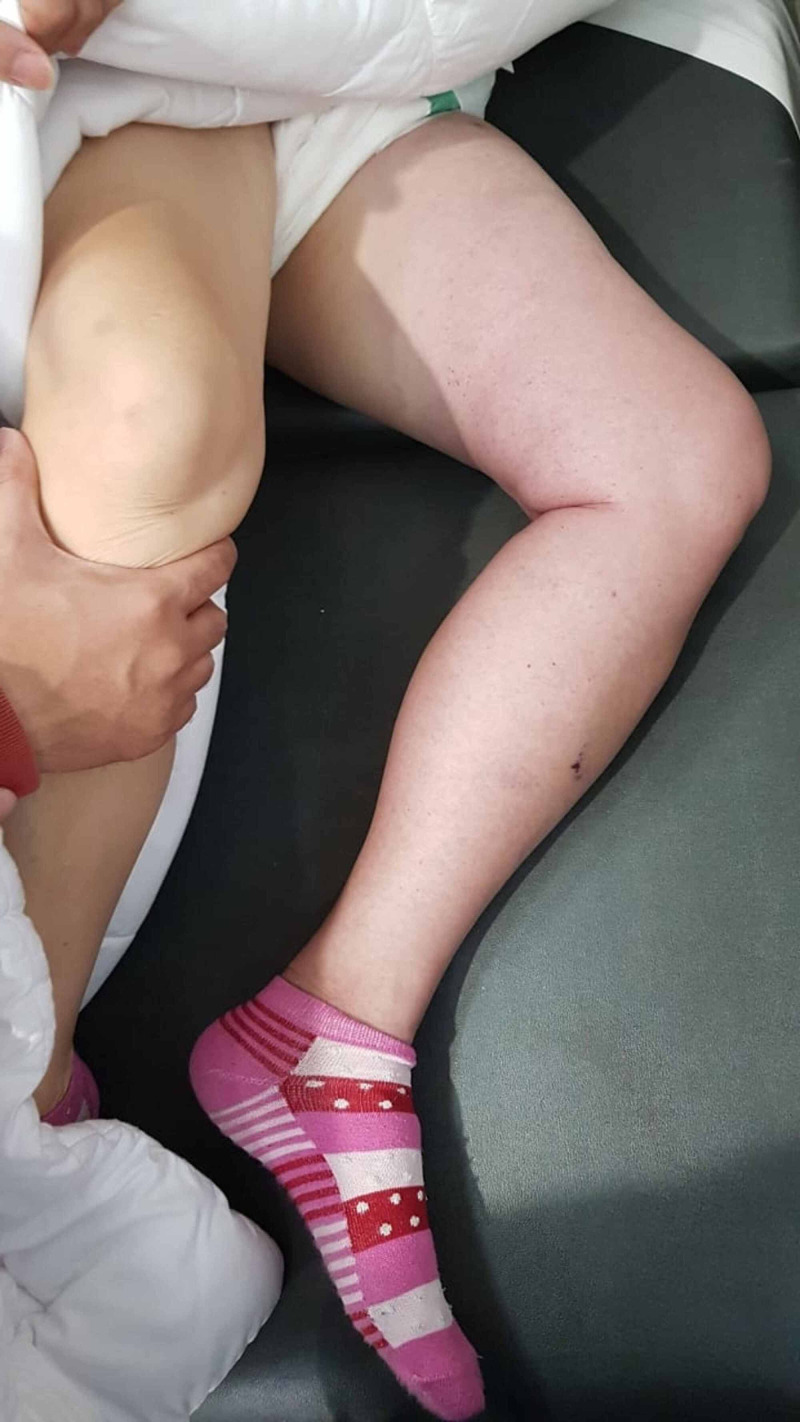
Initial presentation with whole left leg oedema with pale skin and livedo reticularis

**Figure 2:**
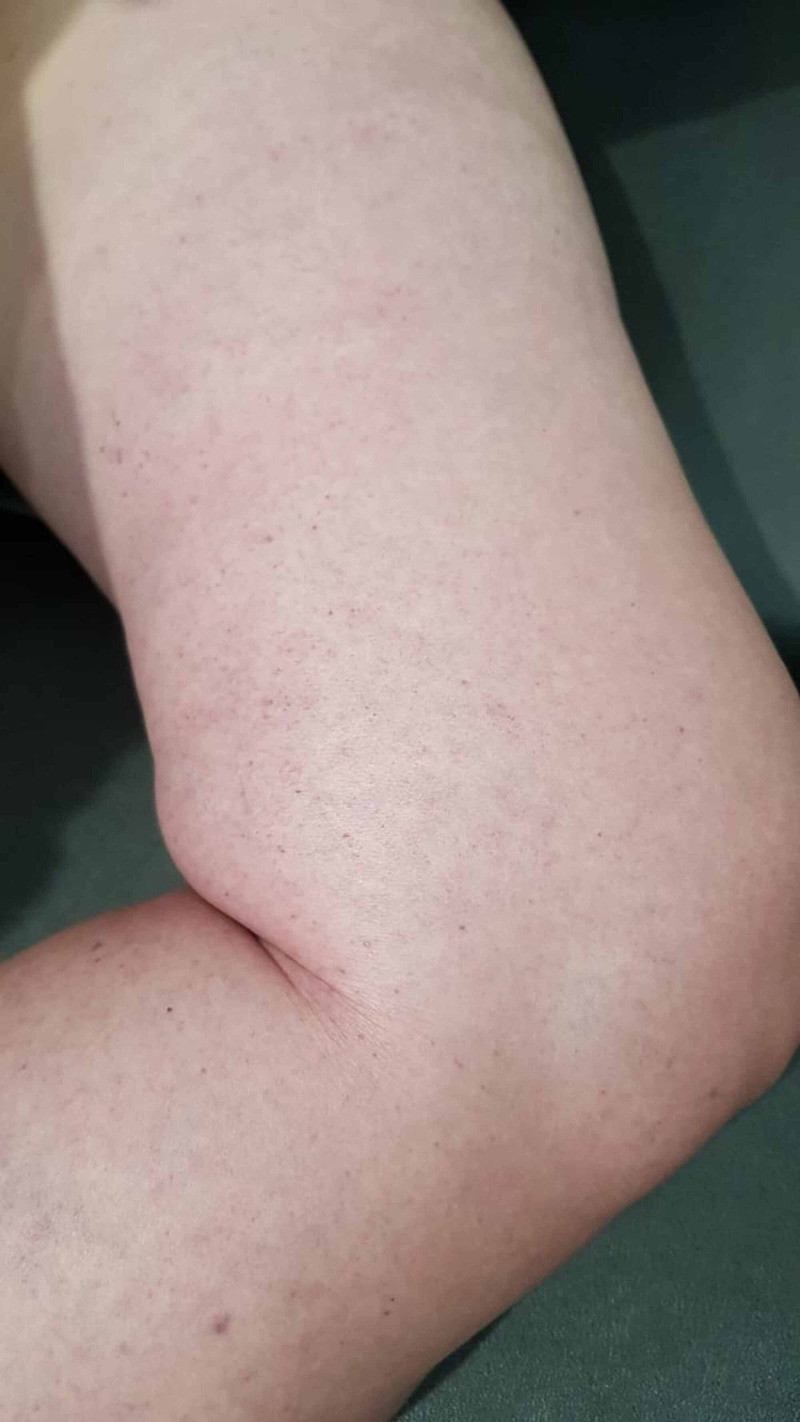
Initial presentation with whole left leg oedema with pale skin and livedo reticularis

A 57-year-old mildly obese woman, known with hypertension and dyslipidemia with poor compliance to medication, presented with sudden onset of excruciating pain at left leg followed by entire leg swelling for 3 hours before visiting our emergency. There was no history of specific provocation or trauma. Before the incidence, she was active and not having any cardiovascular symptoms. She denied taking oral contraceptive agents or other procoagulant medication. On examination, she had tachycardia and mild hypertension, no fever, unremarkable cardiac findings, no palpable abdominal and pelvic masses or bruit. She had significant left lower limb oedema extending up to the upper thigh with pale skin and livedo reticularis (Figures 1, 2). Pulsation of left popliteal and dorsal pedis arteries was very weak. There was poikilothermia and severe tenderness by light touch over the entire left leg and worsened pain by passive dorsiflexion of the left ankle, considered as positive Homan’s sign.

**Figure 3:**
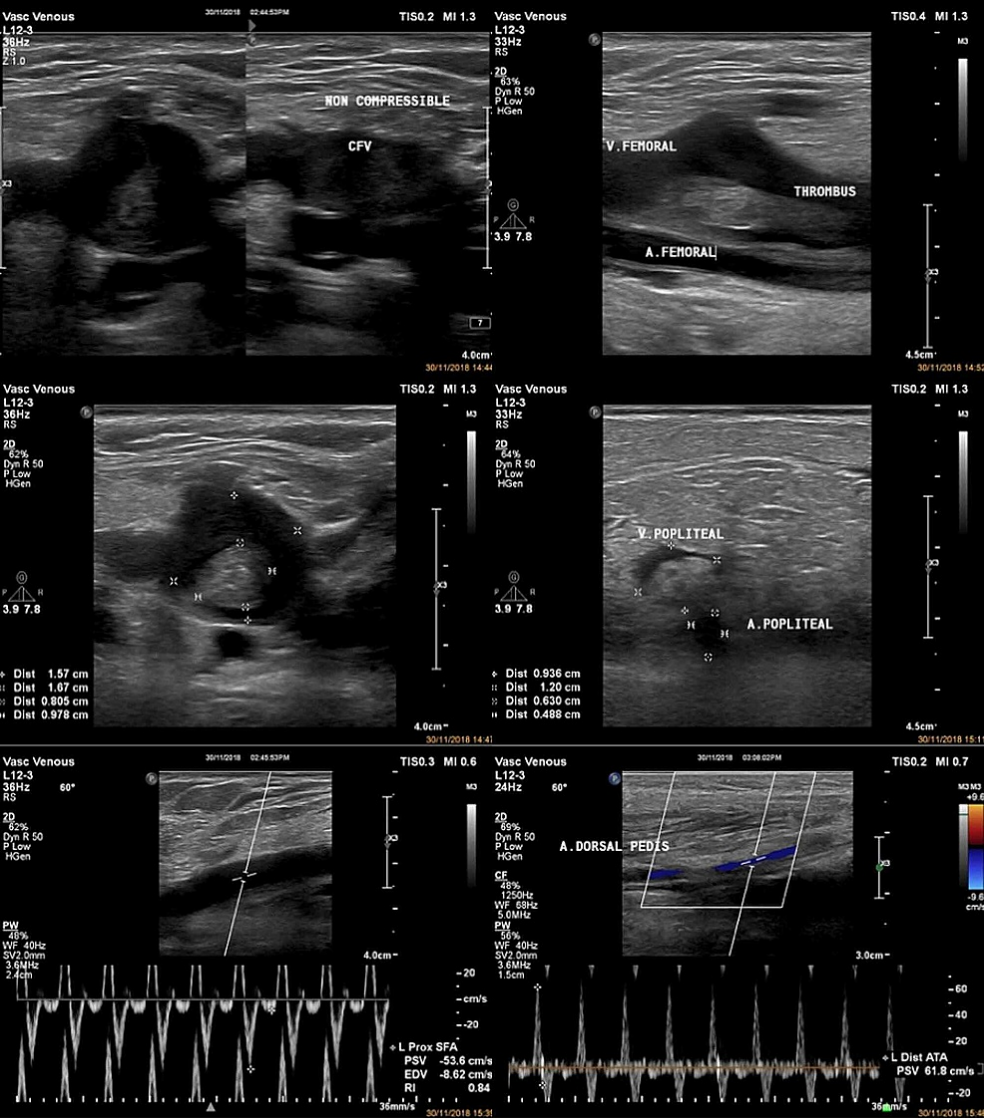
Duplex ultrasound of left lower extremity showed large thrombus located from proximal part of common femoral vein to popliteal vein, along with no flow and non-compressible vein. These findings were consistent to DVT of the leg. Normal flow was noticed throughout arterial systems. DVT: Deep vein thrombosis

Initial blood test showed leucocytosis, D-Dimer 18,000 ng/mL, creatine kinase (CK) level 7256 U/L, creatinine 2.1 mg/dL, C-reactive protein (CRP) >250 mg/L, random glucose level 208 mg/dL, and microscopic hematuria. She claimed of having normal creatinine level in previous medical check-up. Electrocardiography showed sinus tachycardia, left ventricular hypertrophy criteria, and non-specific ST segment changes. Left ventricular hypertrophy was confirmed by further echocardiography examination, besides normal left ventricular systolic and diastolic function. Urgent vascular Doppler sonography of the left lower limbs revealed an obstructive venous thrombus at common femoral vein along with enlarged diameter and non-compressible veins (Figure 3). Arterial systems were normal in diameter without any obliteration, although relatively reduced flow at distal part was noticed. Initial diagnosis was massive deep vein thrombosis (DVT) of left lower limb with rhabdomyolysis and acute kidney injury. The patient was managed with rehydration using saline infusion 2 L daily, bicarbonate orally, anticoagulation using continuous infusion of unfractionated heparin (UFH), and pethidine drip as analgetic.

**Figure 4:**
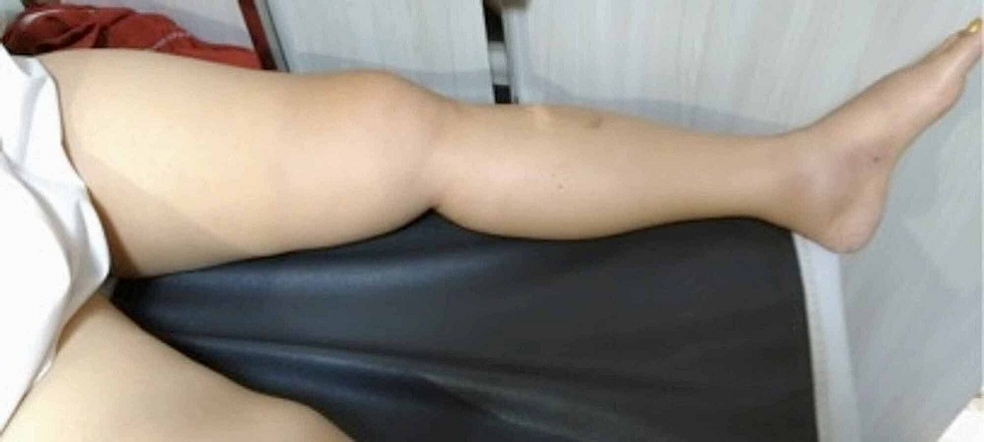
Resolution of left leg edema after treatment for two weeks

**Figure 5:**
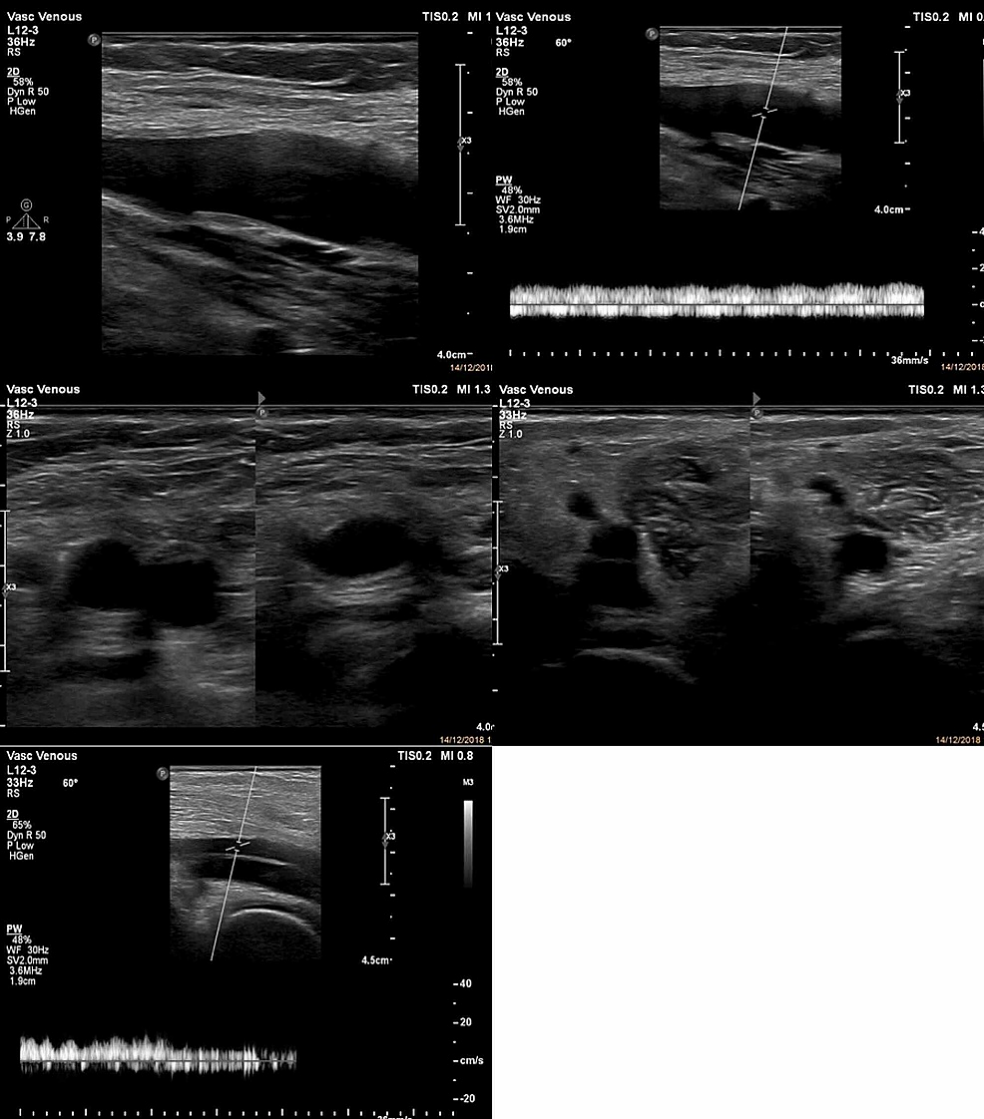
Follow-up duplex ultrasound of left leg after taking anticoagulant for more than one month showed normal flow of vein systems, no thrombus seen, and fully compressible of common femoral vein and popliteal vein.

Non-contrast CT scan of abdomen and pelvis indicated swelling of tissues at femoral region, without detecting any intra-abdominal masses which potentially impeded venous flow. Unfortunately, contrast CT scan was unfeasible to perform due to worsening renal function. Additional work-up for malignancy, hypercoagulable status (including Protein C and S), and autoimmune disorders were negative. Follow-up blood test showed increased creatinine level up to 2.8 mg/dL and CK to 23,393 U/L, and high uric acid level 13.8 mg/dL. Intravenous saline administration and anticoagulation was continued. After treatment for a week, her leg pain and swelling subsided, along with normalization of creatinine level to 1.3 mg/dL and CK to 5651 U/L. Eventually she was discharged with rivaroxaban 2x15 mg and bicarbonate tablet. Later follow-up at clinic in the next week showed no swelling and tenderness in left lower limb, along with normalization of creatinine and CK level (Figure 4). Follow-up duplex ultrasound at the next month indicated significant resolution of DVT, thrombus had diminished and good flow in the venous system had established (Figure 5). Rivaroxaban was continued and it was decided to extend anticoagulation for up to six months, and variceal compression stocking was applied to left lower limb to prevent post thrombotic vein syndrome.

## Discussion

The thrombophlebitic process may go through three phases: phlegmasia alba dolens (milk leg), phlegmasia cerulae dolens (blue thrombophlebitis), and gangrene [4]. Phlegmasia refers to a characteristic clinical picture in which deep vein thrombosis (DVT) causes massive swelling of the entire extremity [7]. Phlegmasia alba dolens is an early phase of thrombophlebitis. This condition is characterized by massive swelling, “white” painful limb, but is not associated with cyanosis. It is observed in patients with iliofemoral deep vein thrombosis (IFDVT) who have enough venous drainage to remove subdermal and dermal venous blood before cyanosis occurs [4,7]. About 44% of phlegmasia alba dolens may develop into PCD and gangrene if not treated and can be life-threatening [2]. The incidence of phlegmasia is unknown due to the rarity of the disease process.

Haimovici reported that etiologic circumstances associated with the cases of thrombophlebitis were postoperative, post-partum, post-traumatic, visceral malignancies, other causes (lung tuberculosis, lung abscesses, cardiac decompensation, bronchopneumonia, post-intravenous infusion), and unknown etiology [4]. In this case, the patient denied any trauma, cancer, hypercoagulable state, prolonged immobilization, hormonal therapy or other procoagulant medication, and was without detected compressive mass in abdomen or pelvis. However, the patient had uncontrolled hypertension, severe dyslipidemia, and obesity. Obesity and age have been reported to play important roles in the risk of uncontrolled hypertension and further complication of thrombophlebitis [8]. Oguzkurt et al. reported that mean age of the patients is 58 (range 31-80) [7]. Studies on the association between thrombophlebitis and hypertension have been reported for several years but there is still controversy. Some studies reported that hypertension could increase the development of thrombophlebitis. Meanwhile, other studies reported that there was no statistically significant correlation between hypertension and thrombophlebitis [8].

Clinical manifestation of phlegmasia alba dolens is characterized by swollen, pale, and painful limb. Swelling can rapidly develop and may extend beyond groin in severe cases. The skin of the affected limb is glossy, exceedingly tense, and the edematous area has a woody consistency [4]. Severe limb oedema can lead to arterial compression and insufficiency flow that may cause severe pain depending on the degree of tissue ischemia [7]. Arterial pulses are still palpable in one-third of the cases, while in the remaining two-thirds, pulsation of distal is diminished. Local temperature is sometimes normal, however, in most cases coolness or poikilothermia of the distal parts of the limb is significant [7]. Left lower extremity is affected four times more often than the right. This is possibly caused by iliac vein compression syndrome, which also may occur with vein thrombosis [8]. In this case, the patient complained of acute swelling and pain of entire left leg. We recognized pale, poikilothermia, swelling, inflammation, and tenderness of the entire left leg, along with weak pulse of left popliteal and dorsalis pedis arteries. These findings were consistent with the clinical manifestation of massive DVT at proximal limb resulting in ipsilateral arterial compression and tissue ischemia, named as PAD [9]. Additional laboratory results of elevated urea, creatinine, and creatine kinase (CK) in PAD raised suspicion of rhabdomyolysis related to compartment syndrome and acute kidney injury (AKI). These conditions were considered as secondary impact, but important to recognize [10]. Rhabdomyolysis is characterized by the triad of muscle weakness, myalgias, and dark urine. However, only about 50% of adult patients with rhabdomyolysis present with the classic triad while other additional symptoms are often not specific. The most reliable and sensitive indicator of muscle injury to diagnose rhabdomyolysis is creatine kinase. CK levels five times the reference range (55-170 U/L in male and 30-135 U/L in female adults) suggest rhabdomyolysis, while CK levels 2-3 times the reference range in patients with risk factors for rhabdomyolysis may indicate early rhabdomyolysis. CK levels rise within 12 hours of muscle injury and peak in 24-36 hours. The peak CK level when it is higher than 15,000 U/L may be predictive of renal failure. Imaging studies, such as magnetic resonance imaging, are the modality of choice for evaluating the distribution and extent of injury of affected muscles, especially when fasciotomy or involvement of deep compartments is considered. The overall mortality for patients with rhabdomyolysis is approximately 5%, however, the prognosis may worsen based on the severity of AKI and extremely elevated CK levels, different underlying etiology, and the presence of comorbid factors [11]. In this patient, the ischemia and rhabdomyolysis of the leg were subsided after given optimization therapy with anticoagulation for massive DVT.

The main causative factor in phlegmasia is massive thrombosis and occlusion of major venous channels with significantly compromised venous outflow [5]. Obstruction to venous outflow leads to changes in the forces that govern influx and outflow of fluid at both the arterial and venous end of the capillary. Under the normal Starling equilibrium, hydrostatic pressure drives fluid out of the capillary into the interstitium at the arterial end and fluid is reabsorbed at the venous end where colloid oncotic pressure exceeds hydrostatic pressure [12]. When the venous outflow is occluded, hydrostatic pressure at the venous end of the capillary rises to exceed colloid oncotic pressure and interstitial oedema develops [3]. In PCD, the thrombosis extends to collateral veins, resulting in venous congestion with massive fluid sequestration and more significant oedema [5]. Following occlusion or near occlusion of venous drainage of an extremity, there is a rapid rise in venous and capillary hydrostatic pressures. Venous pressure may increase 16-fold to 17-fold within 6 h [5]. Fluid sequestration in the interstitium may reach 6-10 L in the affected extremity within days and results in hypovolemia, hemodynamic instability, and a rise in interstitial tissue pressure [5,13]. If untreated, increased interstitial pressure and hypovolemia lead to arterial collapse, distal tissue ischemia, and venous gangrene. Development of tissue necrosis and venous gangrene is a late sign and the prognosis and response to therapeutic intervention significantly worsens; thus, early therapeutic intervention is designed to prevent the onset of venous gangrene by relieving venous hypertension and increasing distal tissue perfusion [3].

Early anticoagulant administration is pivotal step in treating patients with massive DVT. Unfractionated heparin was chosen in this case as she had significant renal dysfunction on initial presentation, hampering the use of low-molecular-weight heparins (LMWH), fondaparinux, or direct oral anticoagulant (DOAC) [14-15]. Leg elevation during lying on bed can alleviate edema. Intravenous resuscitation is needed to prevent intravascular fluid depletion secondary to venous pooling, and also to increase renal perfusion to excrete debris product of rhabdomyolysis. Urine alkalinization by adding bicarbonate can be considered to optimize the later purpose [14].

Advanced procedures are reserved if clinical improvement is suboptimal with aforementioned medication. Fasciotomy and surgical decompression are performed to overcome compartment syndrome, to prevent further muscle necrosis [16]. Endovascular intervention with catheter-directed thrombolysis is usually an effective therapy for the extensive DVT such as in PAD and PCD [10,17]. In rarer cases, mechanical thrombolysis using aspiration thrombectomy devices or balloon angioplasty can be performed. Open surgical thrombectomy is an alternative option for patients with contraindication to anticoagulant or thrombolytic agents, or if these medications are failed to treat the disease. Further placement of an inferior vena cava (IVC) filter to reduce the risk of pulmonary embolism is controversial because of many complications with no significant benefit. Patients with DVT treated with anticoagulants are not recommended for routine placement of IVC filter because of no significant benefit regarding PE or mortality; some data suggest that it may increase the risk of recurrent DVT [9,18-19].

## Conclusions

Rhabdomyolysis is a potential life-threatening complication in massive deep vein thrombosis, even at the initial presentation of this thromboembolic event. Early recognition of this life-threatening situation is crucial to guide the therapy and avoid further systemic derangement. Parenteral anticoagulant is the mainstay in treating this condition, as well as some advanced interventions may be needed in particular cases.
